# 吉西他滨治疗RRM1阴性晚期难治非小细胞肺癌患者1例

**DOI:** 10.3779/j.issn.1009-3419.2011.06.14

**Published:** 2011-06-20

**Authors:** 美玲 赵, 海虹 杨, 建行 何

**Affiliations:** 1 510120 广州，广州医学院第一附属医院，广州呼吸疾病研究所心胸外科 Department of Cardiothoracic Surgery, Guangzhou Institution of Respiratory Disease, the First Afliated Hospital of Guangzhou Medical College, Guangzhou 510120, China; 2 510515 广州，南方医科大学研究生院 Graduate College of Southern Medical University, Guangzhou 510515, China

目前吉西他滨/铂类方案是晚期非小细胞肺癌（non-small cell lung cancer, NSCLC）的标准治疗方法之一。Anderson等^[1]^报告吉西他滨单药治疗NSCLC客观缓解率（objective response rate, ORR）为20%；国内管忠震等^[2]^报道吉西他滨联合顺铂方案的疾病控制率为56.1%，部分缓解率为43.9 %，中位缓解时间（174 ±18.5）天。核糖核苷酸还原酶1（ribonucleotide reductase M1, RRM1）是肿瘤抑制基因，也是吉西他滨的分子靶点。RRM1 mRNA高表达导致肿瘤细胞DNA损伤修复能力增强，从而对吉西他滨/铂类方案产生耐药，RRM1 mRNA低表达提示对吉西他滨敏感^[3]^。基因mRNA表达和蛋白表达有一定的相关性，因此我们采用半定量免疫组化技术检测肿瘤组织RRM1的蛋白表达，现将1例吉西他滨（健择）反复治疗的难治性晚期NSCLC患者的治疗情况及对初诊获得的肿瘤组织进行RRM1等蛋白的免疫组化检测报告如下。

## 1 资料与方法

### 1.1 病例资料

患者，女性，74岁，2006年3月因“反复咳嗽、咳痰1月”入院。胸部CT示右下肺中央型肺癌，并右上肺转移，癌性淋巴管炎。腹部CT、头颅MRI和骨ECT检查无异常。纤支镜病理活检示低分化鳞癌。诊断：“右下肺低分化鳞癌T4N2M0（Ⅲb期）”。随后进行吉西他滨、紫杉醇、长春瑞宾和培美曲塞等铂类联合方案化疗多次（表 1）。治疗反应率根据RECIST标准评估，治疗前后以胸片或胸部CT的影像学资料进行评价。

**1:** 化疗方案及疗效总结表 Chemotherapy and ORR

No.	Time	Chemotherapy	Cycles	Regimen	Adverse events	RR	PFS	Follow-up
1	2006.03-2006.05	GP	2	Gemcitabine 1, 250 mg/m^2^ Cisplatien 75 mg/m^2^	rash, 1 grade leukocytopenia	PR	8 months	To refuse chemotherapy, and receiving TCM
2	2006.12-2007.01	TC	2	Taxol 120 mg/m^2^ Carboplatin AUC=5	1 grade nausea and vomiting, 2 grade leukocytopenia, fatigue	PD	-	-
3	2007.02-2007.04	NP	2	Navebine 25 mg/m^2^ Cisplatien 75 mg/m^2^	2 grade nausea and vomiting, 2 grade leukocytopenia, fatigue	PD	-	-
4	2007.05-2007.08	Gefitinib	3 months	Gefitinib 250 mg/d	rash	SD	3 months	-
5	2007.09-2008.02	GP	5	Gemcitabine 1, 250 mg/m^2^ Cisplatien 75 mg/m^2^	1 grade leukocytopenia, 1 grade anemia	PR	9 months	Disease progression until July, 2008
6	2008.08-2008.09	Alimta	2	Alimta 500 mg/m^2^	1 grade leukocytopenia	PD	-	-
7	2008.10-2008.12	Erlotinib	2 months	Erlotinib 150 mg/d	none	PD		Best support treatment after progression
8	2009.04-2009.06	GP	3	Gemcitabine 1, 250 mg/m^2^ Cisplatien 75 mg/m^2^	1 grade nausea, 2 grade leukocytopenia 1 grade anemia	PR	6 months	Chemotherapy termination because of poor endurance
9	2009.09-2009.12	GP	3	Gemcitabine 1, 250 mg/m^2^ Cisplatien 75 mg/m^2^	1 grade nausea and vomiting, 2 grade leukocytopenia, 2 grade anemia	SD	3 months	-
RR: response rate; PFS: progression free survival; PD: progression disease; PR: partial response; SD: stable disease.								

### 1.2 免疫组化检测

对患者2006年初诊时的石蜡病理活检组织进行相关药敏蛋白免疫组化检测。采用SP法检测病理切片中的ERCC1、RRM1、Beta-Tubulin、TS和BRCA1表达。ERCC1、RRM1和Beta-Tubulin一抗购自北京中杉金桥公司；TS和BRCA1一抗购自DAKO公司。免疫组化染色根据试剂说明书操作。结果评估：RRM1、ERCC1以肿瘤细胞核出现棕黄色颗粒着色为阳性，Beta-Tubulin、TS、BRCA1以肿瘤细胞浆出现棕黄色颗粒着色为阳性。免疫组化标准：每例标本在400倍视野下随机选取500个-1, 500个肿瘤细胞，观察阳性细胞染色强度，并计数阳性细胞百分数。按细胞染色强度记为0分-3分：无着色为0分，淡黄色为1分，棕黄色为2分，深棕色或棕褐色为3分。按阳性细胞百分数的构成比记为0分-3分： < 10%为0分，10%-25%为1分，26%-50%为2分，>50%为3分。以染色强度记分和阳性细胞百分数记分相加所得总分进行判断，总分0-1分为阴性（-），2-3分为弱阳性（+），4-5分为中阳性（++），6分为强阳性（+++）。

## 2 结果

### 2.1 化疗疗效及不良反应

化疗疗效及不良反应见表 1和图 1。患者前后9次的化疗中吉西他滨联合顺铂（gemcitabi-ne plus cisplatin, GP）方案化疗4次，疗效有3次为部分缓解（partial response, PR），1次为疾病稳定（stable disease, SD）。患者GP方案化疗前地塞米松做预处理，化疗中常规应用5-HT3受体拮抗剂止吐，恶心、呕吐等胃肠反应不明显。化疗后骨髓抑制多为1-2度，经休息后2周内能恢复正常，其它不良反应主要为轻度皮疹。

**1:**
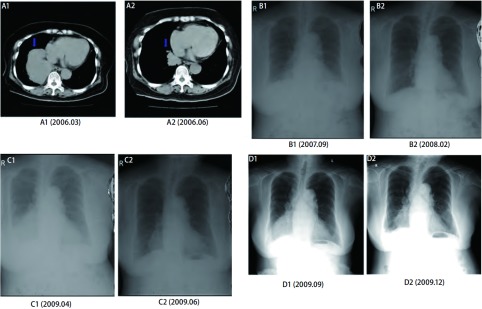
治疗前后CT及X光影像。A1：GP方案治疗前右肺肿瘤（9.6 cm×5.3 cm）；A2：GP方案治疗后右肺肿瘤（4.2 cm×2.2 cm）；B1：GP方案治疗前右肺肿瘤（3.0 cm×2.5 cm）；B2：GP方案治疗后右肺肿瘤几乎消失；C1：GP方案治疗前右肺肿瘤（7.0 cm×8.5 cm）；C2：GP方案治疗后右肺肿瘤（4.2 cm×5.7 cm）；D1：GP方案治疗后右肺肿瘤（6.7 cm×6.5 cm）；D2：GP方案治疗后右肺肿瘤（4.9 cm×6.1 cm）。 CT image and X-ray image before or after chemotherapy. A1: Tumor in right lung (9.6 cm×5.3 cm) before GP treatment; A2: Tumor in right lung (4.2 cm×2.2 cm) after GP treatment; B1: Tumor in right lung (3.0 cm×2.5 cm) before GP treatment; B2: Tumor in right lung (almost disappeared) after GP treatment; C1: Tumor in right lung (7.0 cm×8.5 cm) before GP treatment; C2: Tumor in right lung (4.2 cm×5.7 cm) after GP treatment; D1: Tumor in right lung (6.7 cm×6.5 cm) before GP treatment; D2: Tumor in right lung (4.9 cm×6.1 cm) after GP treatment.

### 2.2 免疫组化检测

RRM1（-）（图 2），ERCC1（+），BRCA1（-），Beta Tubulin（+++），TS（++）。

**2:**
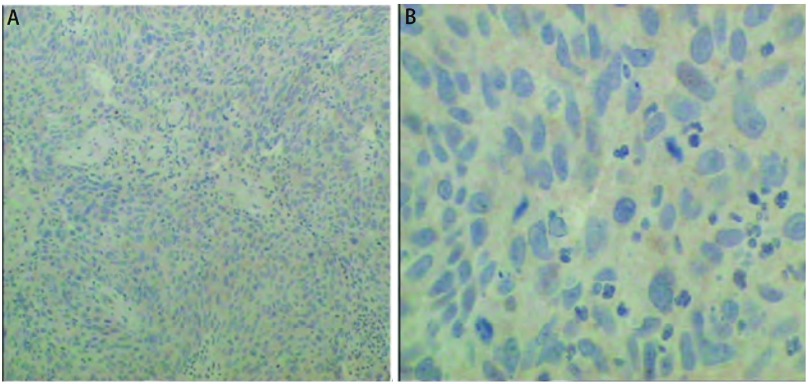
RRM1蛋白免疫组化结果图像。A（SP, ×100）与B（SP, ×400）免疫组化RRM1蛋白低表达。 RRM1 protein expression by immunohistochemistry. A (SP, ×100) and B (SP, × 400) showed RRM1 protein expression negative by IHC.

## 3 讨论

RRM1 mRNA在肺鳞癌中高于肺腺癌，但在不同分期中表达无差异^[4]^。对于晚期不可手术的RRM1 mRNA低表达的NSCLC患者，应用吉西他滨治疗预示着较好的生存期和疗效^[5, 6]^。本研究应用免疫组化技术回顾性检测患者肿瘤组织RRM1等蛋白表达，其中肿瘤组织RRM1表达阴性，4次使用吉西他滨，3次疗效达到PR，1次为SD；无疾病生存（progression-free survival, PFS）时间为3个月-9个月，平均为6.3个月。因此，我们将扩大样本量研究吉西他滨治疗RRM1蛋白免疫组化低表达的NSCLC患者的有效率。研究^[7, 8]^已显示在晚期NSCLC患者中Beta-Tubulin蛋白高表达者对紫杉类药物耐药，TS mRNA高表达者与培美曲塞耐药相关。免疫组化检测患者肿瘤组织Beta-Tubulin和TS蛋白均为高表达，并且患者应用抗微管类药物（长春瑞宾、紫杉醇）及培美曲赛治疗疗效不佳，与上述研究结果相符。

本次报道病例在病情进展后多次使用原来的吉西他滨联合铂类方案治疗，仍获得较好的疗效，提示患者肿瘤进展以后可以考虑重复使用原先有效的化疗方案。吉西他滨联合顺铂化疗方案治疗晚期NSCLC患者毒副反应较轻，患者耐受性较好^[9]^。患者虽为74岁高龄，但对多次的GP方案化疗耐受性较好，并且最佳的疗效是原发病灶消失，转移灶较前明显缩小。由于该患者GP方案化疗疗效佳，吉西他滨的毒副反应轻，耐受性好，是否适宜进行吉西他滨单药维持治疗有待进一步探讨。

目前，关于晚期NSCLC患者接受吉西他滨维持治疗仍有争议。Brodowicz等^[10]^报道接受GP方案化疗，治疗达到缓解后进行吉西他滨维持治疗疾病进展时间（time to progress, TTP）及总生存期（overall survival, OS）明显延长。但是2010年ASCO会议有学者^[11]^报道吉西他滨联合卡铂（gemcitabine plus carboplatin, GC）方案化疗，治疗达到缓解后吉西他滨（1, 000 mg/m^2^, d1, d8）维持治疗并无获益。上述两项研究结果存在差异，可能的原因包括：①维持治疗前化疗方案的铂类不同；②维持治疗的吉西他滨用量不同；③入组进行维持治疗的PR及SD患者构成比不同。上述原因都可能会对结果产生一定影响。此外ASCO会议也有报道^[11]^显示，接受GP方案化疗缓解后进行吉西他滨维持治疗或者厄洛替尼维持治疗对比最佳支持治疗均有获益，并且吉西他滨维持治疗效果更佳。因此，吉西他滨单药维持治疗仍需进一步探讨，关键可能在适宜人群的选择。

综上所述，对晚期NSCLC患者进行RRM1等相关药敏基因的免疫组化检测可能对化疗方案的选择有一定的临床指导意义。
